# Recovered Tire-Derived Aggregates for Thermally Insulating Lightweight Mortars

**DOI:** 10.3390/ma18081849

**Published:** 2025-04-17

**Authors:** Elhem Ghorbel, Safiullah Omary, Ali Karrech

**Affiliations:** 1 Department of Civil Engineering, CY Cergy Paris Université, 5 Mail Gay Lussac, Neuville-sur-Oise, 95031 Cergy Pontoise Cedex, France; 2 INSA de Strasbourg—ICube Laboratory UMR 7357, 24, Boulevard de la Victoire, 67084 Strasbourg Cedex, France; safiullah.omary@insa-strasbourg.fr; 3 School of Engineering, University of Western Australia, 35 Stirling Hwy, Crawley, WA 6009, Australia; ali.karrech@uwa.edu.au

**Keywords:** crumb rubber, lightweight mortars, mechanical properties, thermal properties, end-of-life tires (ELTs), bond strength

## Abstract

This study explores the innovative use of recovered tire-derived aggregates in cement-based mortars to enhance thermal insulation and reduce environmental impact. The research addresses the pressing global challenge of managing end-of-life tires (ELTs), which are non-biodegradable and contribute significantly to waste management issues. By incorporating crumb rubber from recycled tires into mortars, this study investigates the feasibility of creating lightweight, thermally insulating mortars suitable for building repair and rehabilitation. The primary objective is to develop mortars that minimize structural load, decrease energy consumption in buildings, and promote the recycling of ELTs as a valuable resource. The study focuses on evaluating how varying crumb rubber content affects key properties such as workability, thermal conductivity, compressive strength, and fracture energy. Experimental tests were conducted to assess these properties, with the results indicating that mortars with up to 50% crumb rubber content exhibit improved thermal insulation and meet industry standards for non-structural repair applications. The methodology involved creating eight different mortar mixtures with varying proportions of crumb rubber particles (ranging from 0% to 100%). Each mixture was tested for physical and mechanical properties, including density, workability, air content, setting time, thermal conductivity, and strength. The experimental results showed that as the crumb rubber content increased, the thermal conductivity of the mortars decreased, indicating enhanced insulation properties. However, higher crumb rubber content led to reduced mechanical strength, highlighting the need for a balanced approach in material design. Key findings reveal that the air content of early-age mortar paste increases linearly with the crumb rubber replacement ratio, impacting the hardened behavior by concentrating stresses or facilitating the infiltration of damaging elements. The study also establishes relationships between mortar properties and crumb rubber content, contributing to the development of sustainable construction materials. The environmental benefits of recycling ELTs are emphasized, as this practice reduces the reliance on natural sand, a resource that is the second most consumed globally after water. This study underscores the viability of using crumb rubber from recycled tires in mortars for repair and rehabilitation purposes. The developed mortars, particularly those with 25% to 50% crumb rubber content, show promise as non-structural repair products, offering improved thermal insulation and reduced environmental impact.

## 1. Introduction

Managing tire trash and rubber-based items like rubber belts used in mining, construction, and manufacturing is a significant global environmental concern today. Despite being non-hazardous, tire waste poses environmental and public health risks, as noted by [1]:

- Tires contain natural and petroleum-based rubber, steel reinforcements, textile fibes, sulphur, zinc oxide, and carbon black, and are not biodegradable. Rubber undergoes further processing to make it more durable and resistant, making it harder to decompose and recycle.
- Tires are heavy and bulky, so they take up landfill space. Since there are no other technologies to reduce rubber waste, this trend is expected to continue.
- Illegal tire stacks attract mosquitoes. The mosquitoes spread Zika, malaria, yellow fever, and dengue.
- They are also hard to extinguish in fires. Tire rubbers with increased resistance burn longer due to their higher calorific value. When ignited, tires take longer to extinguish. Additionally, tire fires release CO, SO_2_, and NO_2_ gases.
- Used tires decomposing in landfills may release heavy metals and chemicals that pollute soil, groundwater, and farms.

Disposing of millions of end-of-life tires (ELTs) harms the environment and public health [2]. The World Business Council for Sustainable Development [3] estimates that 1 billion tires, or 17 million tons, become obsolete annually. The escalating issue of ELTs has garnered significant attention in recent years, with numerous studies forecasting a substantial rise in their numbers. According to Azevedo et al. [4], the volume of ELTs is projected to reach 1.2 billion by 2030, a trend also supported by Thomas et al. [5]. These studies underscore the urgency of addressing tire waste, as the growing number of ELTs poses considerable environmental challenges. Further corroborating these findings, Abbas-Abadi et al. [6] discuss the complexities involved in managing ELTs, emphasizing the need for innovative recycling solutions. Similarly, Arulrajah et al. [7] explore the potential of repurposing ELTs in various applications, highlighting the importance of sustainable waste management practices.

In addition to the increasing volume of ELTs, the global market for their management is poised for significant growth. ASD Reports [8] predicts that this market will expand at a compound annual growth rate (CAGR) of 4.87% from 2023 to 2032. This growth reflects a heightened awareness of the environmental impact of tire waste and a growing commitment to developing technologies and strategies for effective ELT management. As the market expands, there is an increasing focus on transforming tire waste into valuable resources, thereby contributing to a more sustainable future.

This research aims to explore the potential of using high volumes of crumb rubber tire waste in mortars for building repair and rehabilitation. By investigating properties such as workability, thermal insulation, and resistance to crack propagation, this study seeks to develop mortars that comply with industry standards and promote the recycling of ELTs. The findings of this research have the potential to contribute to sustainable waste management practices and reduce the environmental impact of tire waste.

## 2. State of the Art and Novelty of This Research

### 2.1. ELT Management: State of the Art

The Tire Industry Project (TIP) collected data from 45 countries, including Argentina, Brazil, China, India, Indonesia, Japan, Mexico, Nigeria, Russia, South Africa, South Korea, Thailand, the United States, and 32 European countries. Every year, over 29.1 million metric tons of ELTs are produced, with a 97% recovery rate, with China, the United States of America, and Europe leading in ELT recovery. Furthermore, 25.6 million tons of ELTs are recycled, excluding those used in civil engineering and backfill projects. Table 1 depicts ELT data collected in China, with the specific end use unknown according to WBCSD reports [9]. A total of 88% of ELTs produced are processed rising to 90% when civil engineering and backfill applications are considered. Energy and material recovery are the main ELT recycling methods. Recent reports indicate low recovery rates in ELT management in Argentina, Mexico, Nigeria, South Africa, Thailand, and Russia [10,11].

Table 1 shows the distribution of ELTs in the top tire-consuming nations, according to recent World Business Council for Sustainable Development data [9,12]. Table 1 highlights significant variations in ELT generation and recovery rates across nations, with China and the United States being major contributors. Recovery methods include energy recovery, often through incineration in countries like the United States and Japan, material recycling, which is more prevalent in Europe, and civil engineering applications. The latter involves using ELTs in road construction or as aggregates in concrete, a practice gaining traction in countries like India and Brazil, where ELTs are successfully integrated into construction materials. This approach reduces landfill waste and enhances material properties such as flexibility and thermal insulation.

Despite these advancements, many regions still face challenges due to inadequate infrastructure, lack of regulatory frameworks, and limited public awareness, which hinder the adoption of sustainable ELT management practices. International collaboration and knowledge sharing are essential to promote best practices and innovative solutions. By leveraging successful strategies from countries with high recovery rates, other nations can enhance their ELT management systems and contribute to global sustainability efforts. The data in Table 1 effectively represents the current status of ELT management, highlighting both achievements and areas for improvement, and underscores the potential of using ELTs as aggregates in construction, aligning with sustainable development goals. Continued investment in research, policy development, and international cooperation is crucial for more effective and environmentally responsible ELT management practices.

In the Middle East, millions of tires are disposed of annually, posing a significant challenge. As a temporary measure, these wastes have been stored in urban facilities, illegally disposed of in remote areas, or left in landfills [13]. About 50 million tires are presently disposed of in landfills in Kuwait, and an additional 3 to 3.5 million are thrown away annually. Because of the rough roads and intense heat, tires need to be replaced every two years, which increases waste production [14].

Developing countries struggle to manage solid waste, including ELTs, with few statistics. These factors vary by country and contribute to the lack of waste collection, sorting, recycling, and recovery policies, regulations, and infrastructure. Another reason for tire waste is the lack of regulation on tire import quality, which leads to the importation of second-hand tires (tire retreads) with a short lifespan. Due to this, ELTs in developing countries accumulate in waterways and on land, posing health and environmental risks. Uncontrolled open dumping and burning are the main ELT treatment and disposal methods. This waste management issue is especially severe in rapidly urbanizing nations [15,16,17].

In some African countries, researchers have recently attempted to investigate recycling and recovery options for used tires. Ethiopian cement kilns use shredded ELTs as Tire-Derived Fuel (“TDF”) instead of coal for pre-calcining [18]. Cameroon uses ELTs to make O-rings with market-competitive properties [19]. However, these initiatives remain small.

Due to poor import tire quality control, ELT production is rising in the 14 Pacific Island countries and Timor-Leste (PIC). Like in Africa, used tire imports have a short lifespan and generate lots of scrap. About 22,000 tons of tires are illegally stored, buried, or burned, or 670,600 tires per year. ELT management is lacking due to the lack of specific legislation, except for occasional references to tire burning as a measure to combat atmospheric pollution in some countries (Palau, Fiji, Kiribati, and Cook Islands). In the region, only Samoa restricts imports. PNG, Majuro Atoll, Palau, and Samoa are conducting small-scale shredding operations and studies. Shredded tires can be used as clinker in PNG’s cement kilns and as fuel [20].

Australia generated 450,000 tons of waste tires, or 56 million equivalent passenger units (EPU) tires between 2015 and 2020 [21]. The waste stream in Australia contained 48.5 million EPU tires in 2009–2010, compared to 41.8 million in 2007 [22]. Australia classifies ELTs as passenger, truck, and off-road. Cars, motorcycles, caravans, and domestic trailers use passenger tires. Truck tires are made for buses, light and heavy commercial vehicles, cars, trailers, semi-trailers, and firefighting vehicles. Off-road tires “OTRs” are used on agricultural, mining, construction, and demolition equipment. Australia recovers passenger car, bus, and truck tires well, but not “OTRs”. These have consistently low Australian recovery rates below 11%. Unrecovered ELTs are abandoned in pits or landfills, stockpiled, or illegally dumped. Some authors [22,23,24,25] report that almost all passenger and truck tires are recovered, but OTR tires remain low despite an increase since 2009–2010 (Table 2). Australia’s National Waste Policy aims to recover 80% of ELTs by 2030. This goal requires 55–60% OTR tire recovery. To achieve this goal, efforts to enhance the recovery of tire-derived products (TDP) should be intensified.

In France, some sources [26,27] claim that all EOL tires are recovered and reused. In 2021, 567,762 tons of tires were sold, and 572,370 tons of EOL tires were collected and fully recovered (15.3% reused, 35.8% recycled, 46.8% energy recovered, and 2% Civil Engineering). The national market received 477,200 tons of tires in 2020 and only 85% of tires were collected and recovered (44% energy recovery in cement works, 18% material recovery in granulation, and 16% second-hand sales/retreating). The 2021 processing rate is 29.6 points higher than 2020 due to the health crisis. This level of collection allows for a large volume of tires to be processed, hence the sharp increase in processing rate. In contrast, Article L. 541-10-1 (16°) of the Environment Code establishes a broader producer responsibility (EPR) system for tires and specifies that eco-organizations and individual systems must be approved by 1 January 2023. These regulations advise reducing energy recovery. With this regulatory limit and the reconsideration of waste rubber aggregates in synthetic turf, EOL tire recovery strategies must be updated.

In this context, this research work aims to establish new methods for ELT material recovery. It should be pointed out that the use of ELTs for energy recovery, which involves burning tires, should be discouraged, restricted, or even prohibited to promote material recycling in accordance with the waste management hierarchy (energy recovery is at the lower end of the waste hierarchy and can be considered close to disposal).

### 2.2. Recovery of ELTs as Tire-Derived Aggregate for Mortars/Concrete Applications: State of the Art

Tire disposal is difficult due to their slow aging, heavy mass, and non-biodegradability. Reusing crumb rubber is essential for environmental protection. Crumb rubber is used in the automotive industry, sports facilities, asphalt-based road building, shock absorption, and construction structures (e.g., industrial and bathroom flooring, floor tile, foundation waterproofing, …). Recent studies [28,29,30] show that tire repurposing from collection to scrapping poses few health and environmental risks. However, more research is recommended.

Among the successful civil engineering applications, there is an increasing interest in reusing crumb rubber as a substitute for aggregates in cement-based mortar and concrete [31,32,33,34,35]. Fine aggregates have been replaced with rubber particles of different sizes [36]. Rubber aggregates affect workability and water permeability more than fresh density and concrete strength, according to experiments. Rubberized concrete had higher flexibility but lower compressive and tensile splitting strength in another study, which was a positive gain [37]. Similarly, low percentages of rubber substitution (2–12%) resulted in significant reductions in compressive strength and elastic modulus. Nonetheless, experimental evidence showed that substituting aggregates with rubber particles improved the deformability of concrete [38]. Replacing 25% rubber with silica fume decreased compressive strength but did not affect concrete workability [39]. These authors found that partial cement replacement with silica fume increased compressive strength and elasticity moduli (static and dynamic). While partial substitution of fine aggregates with crumb rubber negatively affected concrete’s physical and mechanical properties, rubber size and content positively affected its abrasive and freeze-thaw properties [40]. An increase in porosity was also observed when increasing the rubber content, contributing to a decrease in the mechanical properties [41]. Studies on the use of CR in concrete are more numerous than those on its application in mortar. For instance, da Silva et al. [42] found that recycled tire rubber particles increased cementitious paste micropore volume.

A composite embedding fine crumb rubber reduces the environmental impact of scrap tires and the net weight of structural mortar, which would otherwise be excessive. Reusing rubber and reducing mortar’s natural sand fraction have environmental benefits. Reusing crumb rubber instead of natural sand is a sustainable design innovation. Densities near 1500 kg/m^3^ are achieved with a CR-to-NS replacement ratio of 100%. This paper provides the first measurements up to a 100% replacement ratio since previous studies were limited to 50%. Some studies have examined the fracture energy of crumb rubber concrete [43,44], but more research is needed on mortars with large amounts of rubber.

To summarize, based on the numerous published papers, there are around 675 articles dealing with the recovery of tire wastes in concrete and/or mortar as aggregates (87% of these publications concern concrete while only 13% are on mortars). Moreover, according to some review papers dealing with the state-of-the-art on this theme [45,46,47,48], it appears that existing studies on concrete consider crumb rubber aggregates at percentages less than 30% by volume. Some works deal with replacements greater than 30% [12,49,50,51,52] and only a few studies focus on total replacement [50,51,52,53,54]. In addition, it appears (Figure 1) that most published investigations deal with mechanical properties (very few of them on fracture) and durability (mainly regarding water absorption). Few papers deal with the application of rubberized concrete in the construction industry and most of them are reported in a review paper [55]. The use of rubberized concrete is often motivated by its low density, good sound absorption, good durability against chemical attack, freeze–thaw and chloride ion diffusion, enhanced damping capacity, impact resistance to bending, and toughness.

Few studies have investigated the use of rubber crumb in plasters and mortars or plasters/mortars applied to exteriors, ref. [56] established that it is possible to manufacture exterior walls with rubberized mortar, ref. [57] investigated the effectiveness of alkali-activated used tire rubber mortar in the repair of damaged reinforced concrete beams.

This work aims to examine the potential use of high volumes of crumb rubber tire waste in mortars intended for the repair/rehabilitation of buildings’ concrete walls. To achieve this goal, several properties required for the repair of mortar must be studied. These properties include workability, which determines how easily the mortar can be mixed, applied, and used for surface finishing; adhesion to the substrate, which depends on the mortar’s properties and the substrate’s surface conditions; thermal insulation; and resistance to crack propagation.

## 3. Objectives and Novelty

Existing papers do not comprehensively address all the essential properties of CR mortars required for repair or rehabilitation in accordance with current standards. Despite the extensive body of research into the effects of crumb rubber on certain physical properties, little effort has been dedicated to the applications of rubber-embedding mortar/concrete.

This research aims to develop a new product for repair/rehabilitation that does not generate an excess load on the structure and its foundations. The proposed solution is anticipated to reduce energy consumption in buildings (in this case, heating and air-conditioning) by improving thermal insulation, increase the potential for recycling EOL tires as a “recovery material”, and reduce the use of natural sand, which is currently the world’s second most consumed resource after water.

In this work, we investigate the effects of incorporating CR waste on the properties of cement-based mortars (mixing, workability, setting time, density, thermal conductivity, strength, stiffness, shrinkage, fracture energy, fire safety, and adhesion between the concrete support and CR mortars). In particular, we aim to develop thermally insulating lightweight mortars (TILMs) for repair applications and ensure that they comply with the following standards:

- NF EN 1504-3 defines the classes of products according to their performance: classes R4 and R3 for structural repair and classes R2 and R1 for non-structural repair.
- EN 206-1 prescribes the minimum compressive strength class at 28 days for structural applications (LC8/9 minimum) and its density class from D1.0 ($800\leq \rho \left(\frac{\mathrm{kg}}{m^{3}}\right)\leq 1000)$ to D2.0 $\left(1800<\rho \left(\frac{\mathrm{kg}}{m^{3}}\right)\leq 2000\right)$.
- NF P 18-840 defines the key characteristics for a good repair, namely very good adhesion to the support, mechanical compatibility with existing concrete, controlled shrinkage, permeability, resistance to chemical aggression from carbon dioxide, chlorides, and/or sulphates, and workability. Hence, CR and NS have been characterized by their granulometries, densities, water absorption coefficients, and heat capacities measured using a deferential scanning calorimetry (DSC) test. At the fresh state, mortars were characterized by evaluating their density, workability (to verify the conformity to NF P 18-840), setting time, and air content tests. At the hardened state, the mortar was tested by measuring its density (to check its density class), porosity, three-point bending, and compressive strength (resistance class) to verify the influence of CR on the physical and mechanical performance of mortar. The adhesion of CR mortars to the support, the mechanical compatibility with existing concrete, and the shrinkage were also assessed.

## 4. Materials and Methods

### 4.1. Materials

Semi-crushed natural sand was substituted by crumb rubber aggregates. CR was provided by DeltaGOM, Noyon, France and produced in a recycling platform of non-reusable tires from different categories (including heavy vehicles, passenger vehicles, agrarian, and motorcycles). For all mixes, Portland cement (CEM II/A-L 42.5) and limestone filler (HP-OG supplied by Omya SAS, Omey, France) of respective densities 3.09 and 2.7 g/cm^3^ were used. The latter was instrumental in increasing the mortar viscosity. To ensure high workability, a superplasticizer of type MC-Power Flow-3140 was employed in all developed mixtures.

### 4.2. Experimental Procedures

Granulometry, density, water absorption, and deferential scanning calorimetry tests were carried out to characterize the CR and NS. To determine the size distribution of CR and NS, sieving was conducted according to the French Standard (NF EN 933-1 2009). The density and water absorption coefficient of CR and NS were measured by the pycnometer method according to the French Standard (NF EN 1097-6 2014). As the density of CR is low, the measurement was performed in ethanol.

At the fresh state, the mortar was subjected to workability testing versus time (90 min) by using slump tests performed according to the French standard NF EN 12350-2 (2010). The slump was measured using a special mini cone whose dimensions are deduced from Abram’s cone by a homothetic ratio of two (upper diameter = 50 mm, lower diameter = 100 mm, and height = 150 mm). In addition, the setting time was measured using a penetration resistance test and the air content was measured using an aerometer, according to the French Standard (NF EN 12350-7 2012).

At the hardened state of mortar, the French Standard (NF P 18-459 2010) was applied to determine the density, WA coefficient, and total porosity. These measurements were carried out after exposing the specimens to vacuum for 4 h and immersing them in water for 44 h. The following expressions were used to calculate the coefficient of water absorption and porosity, respectively:

$$WA=\frac{M_{air}-M_{dry}}{M_{dry}}\;\mathrm{and}\;n=\frac{M_{air}-M_{dry}}{M_{air}-M_{w}}$$

where *M_air_* is the mass of aggregates at the saturated surface-dried state, *M_dry_* is the mass of aggregates at oven dried state and *M_w_* is the mass of aggregates in water.

The thermal conductivity and volumetric heat capacity of all mortars were determined according to ISO 22007-2. The samples were previously dried at 60 °C. The tests were conducted on prismatic specimens (4 cm × 4 cm × 16 cm using a hot disc machine based on the Transient Plane Source with 80 mW output power and 40 s measuring time.

Drying tests were performed on prismatic samples cured in water for 28 days after demolding. The samples were then placed in the oven at 60 °C and 40% RH, while the mass was measured continuously during drying for up to 16 days.

Shrinkage tests were performed on prismatic samples demolded 24 h after casting and stored in a room at 20 °C and 50% RH according to the NF P15-433 standard. The length variation of the specimens was evaluated using an apparatus equipped with a reference bar and a digital indicator.

The flexural strength test was carried out on prismatic specimens (40 mm × 40 mm× 160 mm) using an Instron machine of capacity 30 kN according to the French standards (NF EN 196-1 2006). Compressive strength was tested on cubic mortar specimens (40 mm× 40 mm× 40 mm) and was performed using a servo-hydraulic machine of capacity 3500 kN with a loading rate of 0.5 MPa/s.

The dynamic modulus of elasticity (Ed) is estimated based on resonance frequency measurements, according to the standard NF EN ISO 12680-1 using an E-Meter MK II device supplied by James Instruments.

The fracture energy, Gf, was estimated using standard three-point bending tests performed on pre-notched specimens [58]. For each specimen, a mode I crack propagated in the midsection of the beam under the applied load. During this process crack mouth opening displacement (CMOD) and deflection versus applied load were both recorded using linear variable differential transformers (LVDTs). For each experimental point, the test was repeated at least 3 times.

Pull-out tests were carried out according to the EN 1542 standard and the guidelines of the AFGC. A 2 cm layer of mortar was applied to a concrete slab measuring 30 cm × 30 cm × 10 cm and of resistance class C25/30. Seven concrete slabs were elaborated in order to apply the 7 mortar mixes (MCR-0% to MCR-100%). Before applying the repair mortar, the surface of the slabs was cleaned, and its roughness was determined using a volumetric patch and Elcometer 224 gauge. Then, on each slab/mortar 5 circular cracks with a diameter of 50 mm and a depth of 4 mm were performed. A steel cylinder of diameter 50 mm was then glued to the area delimited by the circular crack using epoxy glue. A tensile load was applied to the cylinder at a constant rate of 0.05 MPa/s using a Proceq DY-216 dynamometer, Zurich, Switzerland, until failure occurred. Such a process made it possible to evaluate the concrete/mortar bond strength.

Finally, the effect of heat treatment on the behavior of cured mortars at 28 days of age was investigated using an electrical furnace (of maximum temperature 750 °C and volume 1.35 m^3^) and a TGA testing device (STA 449 F1Jupiter, developed by Netzsch, Selb, Germany).

### 4.3. Characteristics of CR and NS

The size distribution curves (RHS of Figure 2) show that both materials were comparable although CR had slightly fewer fine particles (<2 mm) than NS (Figure 2a). Their fineness moduli (FM) were calculated according to the French Standard (NF EN 12 620 2008) and it can be seen that CR had a greater fineness modulus than NS (Figure 2a). Moreover, microscopic observations underlined that the CR particles had rough surfaces while NS particles were round (Figure 2b).

The densities $\left(\rho_{rd}\right)$ and the water absorption coefficients (WA) of NS CR were determined according to the French Standard (NF EN 1097-6 2014). For sand, the test was carried out in water but for CR it was conducted using an ethanol solution because CR has low density. The experimental results are presented in Table 3 showing that CR exhibits a low density, which can be critical for the mix design, especially in terms of segregation. Moreover; the particle size ratio $R_{s}$ used to quantify the relative size of shredded tire particles (CR) and sand particles with similar shape gradations is calculated ($R_{s}=\frac{D_{50}^{CR}}{D_{50}^{NS}}$ where $D_{50}$ is the particle size corresponding to 50% passing). Several authors [59,60,61,62] have found that the relative size ratio affects granular mixture thermal properties. They found that thermal conduction is better with large insulating aggregates than small ones. They observed that small, shredded tires ($R_{s}<1$) have a greater thermal insulation effect than large particles due to their ability to encircle larger sand particles. In this study, the ratio is slightly greater than 1, indicating that the insulating effect should be effective but not significant.

Batch leaching tests in a neutral medium (leaching with deionized water pH = 7) were carried out according to the NF EN 12457-1 standard to gain information about the constituent concentration release from CR particles. The purpose of this leaching test is to compare the composition of leachates with the threshold values and detect contaminants, which could be mobile in water and could be harmful to the environment.

In this study, the pH of the solution was measured before and after batch leaching using a static pH test. The concentrations of inorganic species leached in water were determined by inductively coupled plasma (ICP) spectroscopy and expressed in mg/L. The results show that the pH values of the influent and leachate are similar (7.5 and 7, respectively). Moreover, the concentrations of leached pollutants are below the limit values set by the Environmental Protection Agency in 2011 (as shown in Table 4). This confirms that the use of these wastes as sand to prepare mortars for building applications is safe and environmentally friendly.

### 4.4. Mortar Mixtures

The mini cone test was conducted to ascertain the appropriate dosage of superplasticizer and to analyze the spread flow characteristics of the cement paste. Figure 3 displays the experimental data for the weight percentage of spreading versus superplasticizer. The spreading increased as the superplasticizer weight increased and leveled off at 2.5%, showing no significant change in cement paste fluidity beyond this point. Figure 3 displays the outcome achieved with plain mortar, where 1.5% of superplasticizer by weight was considered sufficient when CR was incorporated into the paste.

Seven rubber–cement matrix mixes with varying levels of natural sand substitution by crumb rubber were studied. The mortar mixes were formulated by combining Portland cement type I, sand, water, and limestone filler. The design was enhanced by including a reference/control case that did not contain rubber. In this study, the volumetric ratio of substitution, *r_v_*, is defined as:

$$r_{v}=\frac{V_{CR}}{V_{NS}+V_{CR}}$$

where $V_{CR}$ is the volume of crumb rubber and $V_{NS}$ is the volume of natural sand. The rubber contents in the seven mixes were 0%, 10%, 25%, 50%, 60%, 75% and 100%. The mortar compositions are detailed in Table 5. The table labeled the mixes with “M” for mix, “CR” for crumb rubber, and a number indicating the volume percentage of natural sand replaced by CR. The water–cement (W/C) and water–binder (W/B) ratios remained consistent across all mixtures. It is evident that the density of the mortar in its fresh state decreased with higher replacement ratios.

## 5. Results and Discussion

### 5.1. Fresh State Properties of the Mortars

A testing campaign was carried out on freshly prepared mortar to assess its early-age properties such as density, workability, air content, and setting time for each mixture. Density experimental results are presented in Table 5, while air-content and workability results are shown in Figure 4. Figure 4a demonstrates a linear increase in air content with the replacement ratio as shown below:

$$a\left(\%\right)=0.04r_{v}+2.64\;\mathrm{with}\;R^{2}=0.93$$

The total setting time was determined by conducting the penetration resistance test at room temperature (20 °C). Figure 4b displays experimental results indicating that setting time decreases as the replacement ratio increases. The results do not indicate a precise threshold for acceptable performance. However, they offer designers a range of variations in air content and setting time in relation to the replacement ratio, which can be useful for practical applications. The air content varies by approximately 200%, and according to homogenization principles, this level significantly impacts porosity, mechanical properties, and thermal properties, as noted by some authors [64,65]. The rise in air content as the crumb rubber aggregates’ “CR” fraction increases is due to their non-polar nature and rough surface, which traps air bubbles (Figure 2b). A number of researchers have independently verified this phenomenon [66,67].

Figure 5 shows the results of slump testing for various specimens obtained according to NF EN 12350-2 and NF EN 1015-3 standards. It indicates that workability significantly decreases when the CR content exceeds 60%. These results confirm the trend observed in the literature [35,68]. Figure 5 does not specify a particular threshold for workability but offers a range of options to help designers choose the suitable mixture based on the intended application. The EN NF 1015-2 standard recommends a spread of 175 ± 10 mm for repair mortars with a fresh state density exceeding 1200 kg/m^3^, as determined by the EN NF 1015-3 standard. Given that the elaborated mixes (Table 5) have densities greater than 1200 kg/m^3^, mortars with $r_{v}\leq 60\%$ meet these guidelines.

Furthermore, all the mixtures retained their slumps for approximately 20 min. Following this time frame, a significant decrease in the material’s ability to be worked was noted, particularly when the CR content surpassed 60%, as shown in Figure 6. The relative slump in this figure is represented by the equation $S_{sr}=\frac{S_{t}}{S_{i}}$, where S is the designed slump, “t” is the time in minutes, and “i” is the initial slump.

Accordingly, mortars with $r_{v}\leq 60\%$ can be deemed suitable for repairing applications from a workability perspective, in line with standard EN NF 1015-3 recommendations.

In conclusion, the fresh state properties of the mortar, such as density, workability, air content, and setting time, are critical for its application in construction. The linear increase in air content with the replacement ratio of crumb rubber (CR) indicates that CR particles, due to their non-polar nature and rough surface, trap air bubbles. This increased air content can significantly impact the porosity and mechanical properties of the hardened mortar. The decrease in setting time with higher CR content suggests that the mortar may set faster, which could be beneficial for rapid construction but might also limit the working time for application.

### 5.2. Hardened State Properties of the Mortars

a. Density and porosity

The density of various hardened mortars was determined by exposing the specimens to vacuum for 4 h and then immersing them in water for 44 h. The experimental results are graphically depicted on the left-hand side of Figure 7. The mortar density decreased as the ratio of crumb rubber to natural sand increased because rubber has a lower density compared to sand. Substituting NS with CR completely (MCR-100%) decreased the overall density by 50% compared to the control mortar [68,69,70]. The experimental results aligned with the literature [24,54,61,69] as shown on the right side of Figure 7. Our experimental results included a full range of measurements from 0% to 100% replacement ratio, unlike previous research that only covered up to 50%. The results indicated that density decreased as the replacement ratio increased, following an established equation:

$$\rho_{ap}\left(g/{\mathrm{cm}}^{3}\right)=2.17e^{-0.01r_{v}}\;\mathrm{with}\;R^{2}=0.88$$

Furthermore, it should be noted that for $r_{v}\geq 25\%$ the mortar can be considered as light because its density is less than 1900 kg/m^3^. The standard EN 206-1 prescribes for lightweight concrete a range of density varying from 1800–2000 kg/m^3^ to 800–1000 kg/m^3^ (Classes D1.0 to D2.0). Hence, the mortar can be considered lightweight for $r_{v}\geq 25\%$ with Class D1.0 for MCR-25% and Class D1.2 for MCR-100%.

Figure 8’s left side displays the water absorption results, while the right side shows the total porosity that is accessible to water, which includes the pore spaces in the cement paste and between the interfaces. The water absorption coefficient, WA, rises as the CR content increases. When NS was completely substituted with CR, WA showed a significant rise. These findings align with the existing literature [65,71,72,73]. The following equation provides a good description of the increase in WA in relation to the CR replacement rate $r_{v\;}\left(\%\right)$:

$$WA\left(\%\right)=7e^{0.01r_{v}}\;\mathrm{with}\;R^{2}=0.89$$

Likewise, the mortar’s porosity rises as the CR content increases. The growth is gradual when $r_{v}\leq 50\%$, but it becomes substantial otherwise. The results obtained align with the existing literature [39,71]. The rise in porosity is commonly linked to various factors such as the water-to-cement ratio [39,73] or the entrapment of air by rubber particles during mixing [73]. Additionally, poor adhesion between rubber particles and the mortar matrix, particularly with CR spheroid particles, can exacerbate this phenomenon [24,74]. The water-to-cement ratio in this research was kept at 0.55, which differed from previous studies. Furthermore, it is crucial to note that past investigations were restricted to replacement ratios of less than 50%, typically using fine and coarse rubber aggregates. We explore a complete range of replacement ratios (from 0 to 100%) to achieve more thorough results in our research. Furthermore, we concentrate on fine rubber crumb in order to prevent long interfacial transition zones. Decohesion between the waste crumb rubber particles and the cement matrix is not observed by SEM (Figure 9). Furthermore, the sample’s EDS shows that the interface is thin in relation to the grain sizes. The variation in elemental concentrations can be used to deduce this. For instance, the figure begins with a large concertation of C, indicating the presence of a rubber particle up to 300 μm, and moves right to left. A cementitious interface of roughly 50 μm is indicated by the concentration of Ca increasing and the concentration of C decreasing between 300 and 350 μm. Furthermore, Figure 9 demonstrates that the concentrations never reach zero at the same time, indicating that the specimen does not contain major pore spaces—at least not along the path depicted in the figure. Therefore, it is not possible to attribute the rise in mortar porosity to the rubber particles’ debonding from the cement matrix. On the other hand, the presence of microcracks and tiny voids at the transition zone could be the cause of the porosity increase [74,75].

Hence, it can be concluded from this study that the increase in porosity is mainly attributed to the higher occluded air content associated with higher CR rates. This phenomenon is particularly pronounced for $r_{v}>50\%$ (Figure 10).

The evolution of density versus porosity is shown on the left side of Figure 11. When the replacement ratio rises, it does as expect. This study establishes a relationship between both properties based on literature works and experimental results:

$$\rho_{ap}\left(g/{\mathrm{cm}}^{3}\right)=3e^{-0.93n\left(\%\right)}\;\mathrm{with}\;R^{2}=0.82$$

In a comparable manner, an increase in WA occurs when the porosity increases, as shown on the right side of Figure 11. The evolution is expressed based on the results of the current study and previous literature [74,76]:

$$WA\left(\%\right)=2.19e^{0.09n\left(\%\right)}\;\mathrm{with}\;R^{2}=0.93$$

b. Thermal properties

The results obtained indicate that as the CR content increases, the thermal conductivity of the mortars decreases. This indicates that for a given thickness, the mortar has a greater capacity to resist cold and heat (Figure 12a). Higher thermal resistance (R=e/λ) indicates better insulation properties in a product. Shredded tire particles are almost adiabatic with $0.193\leq \lambda \left(W/\mathrm{mK}\right)\leq O.213$ [57,64] and a value of $\lambda \left(W/\mathrm{mK}\right)=0.25$ according to [61]. In comparison, natural sands exhibit higher thermal conductivity, typically ranging from 1.8 to 3.6 W/mK, influenced by factors like chemical composition (especially quartz), grain size, porosity, and saturation level [61,77]. The authors assert that the thermal conductivity of sand primarily composed of quartz runs within the range of 3 to 8 W/mK [59]. Thermal conductivity of tire crumb rubber particles, “CR”, is consistently at least ten times lower than that of natural sand. As a result, as the ratio “CR” increases, rubberized mortars’ thermal conductivity decreases. This reduction is amplified when the relative particle increases. Figure 12a clearly shows an 82% reduction in thermal conductivity between the reference mortar and the one containing 100% CR.

The thermal inertia of mortars, a term widely used to describe the ability of a material to store heat, is characterized by the diffusivity “a“ $\left(a=\frac{\lambda }{C}\right)$ and the effusivity “e” $\left(e=\sqrt{\lambda C}\right)$. Both properties are dependent on the volumetric heat capacity (Figure 12b) and thermal conductivity (Figure 12a).

Diffusivity measures mortar–environment thermal energy exchange. Therefore, optimal comfort necessitates low diffusivity to ensure that this exchange occurs at a slow rate. The rise in the CR content significantly reduced the thermal diffusivity (Figure 12c). For example, the relative decrease in diffusivity is 61% for $r_{v}=60\%$. Regarding temperature variation, effusivity is the predominant property. Hence, the repair/rehabilitation mortar needs to have a high thermal effusivity to effectively store energy and reduce the impact of temperature fluctuations within a structure. The results obtained indicate that the effusivity does not rise as the CR content increases (Figure 12d). The reduction is approximately 20% for 60% CR content and 64% for 100% CR content.

These outcomes indicate that using mortars with up to 60% CR particles enhances comfort and thermal resistance without significantly impacting thermal inertia.

**Figure 13:**
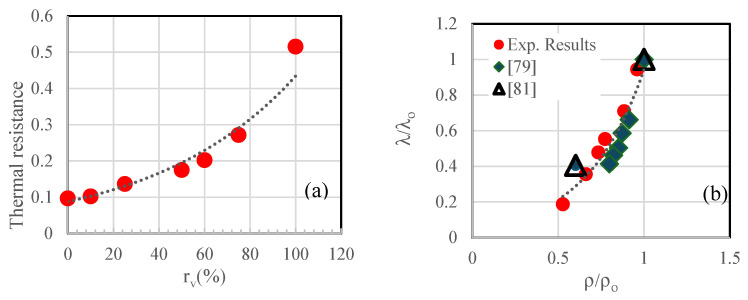
Effects of incorporating tire crumb rubber particles on the thermal properties of mortars (e = 20 cm): (**a**) thermal resistance against the incorporated crumbled rubber tire particles ratio and (**b**) the normalized thermal conductivity against the normalized density of the different mortars [79,81].

**Figure 1:**
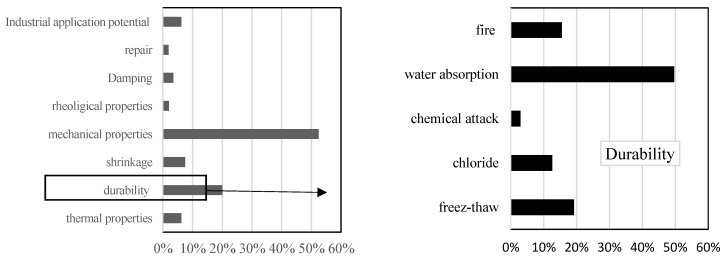
Field of literature studies concerning the recovery of tire wastes in concrete.

**Figure 2:**
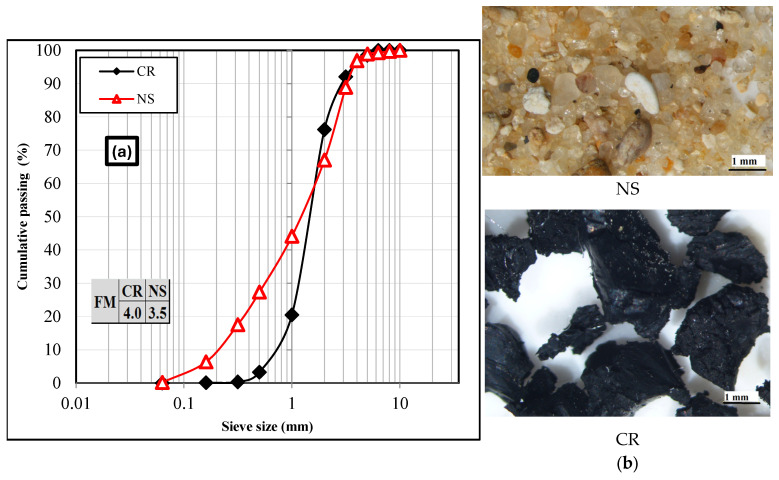
(**a**) Size distribution curves (LHS) and (**b**) microscopic observations (RHS).

**Figure 3:**
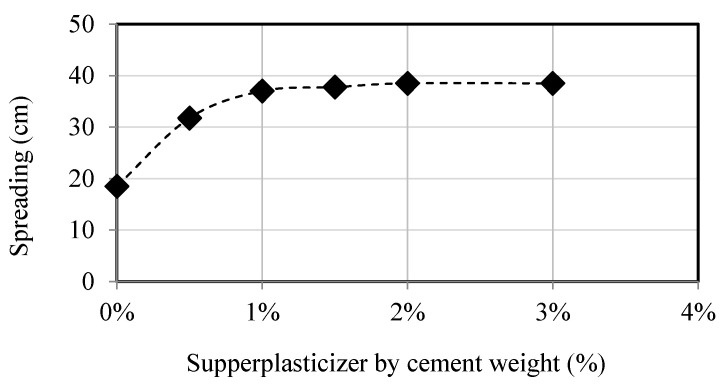
Saturation dosage of the superplasticizer.

**Figure 4:**
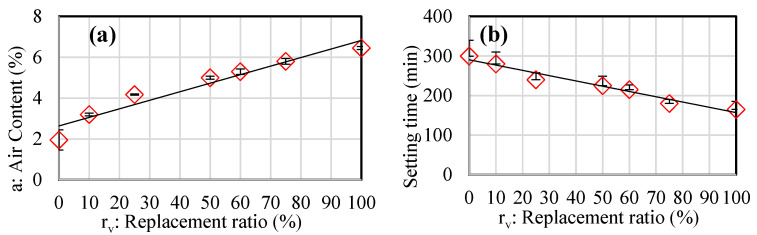
Variation of air content versus replacement ratio and slump of fresh mortar.

**Figure 5:**
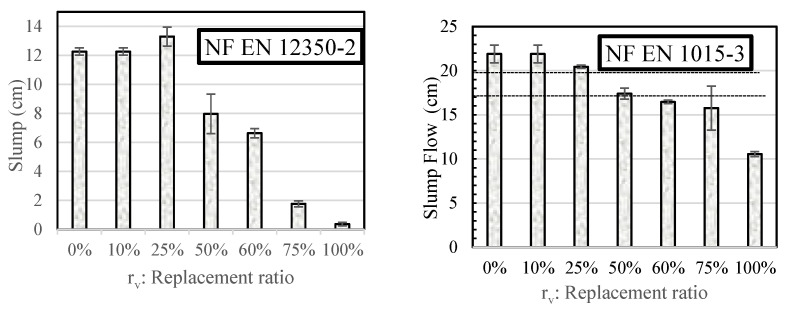
Workability of the different mixes as a function of CR rates.

**Figure 6:**
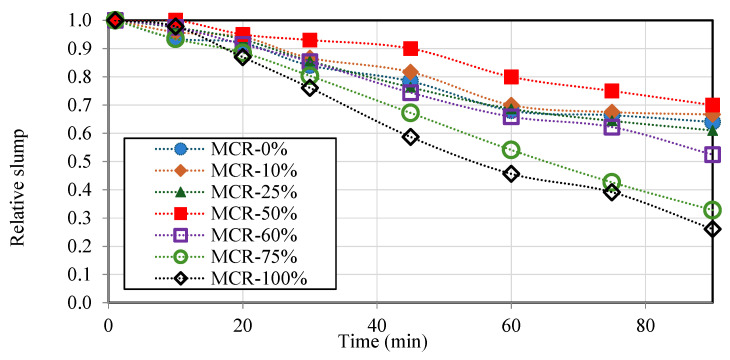
Relative slump of mortar versus time.

**Figure 7:**
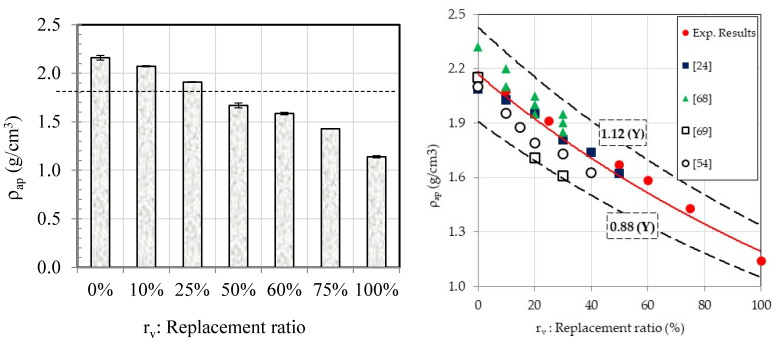
Density and water absorption coefficient of different mortar mixes [24,54,68,69].

**Figure 8:**
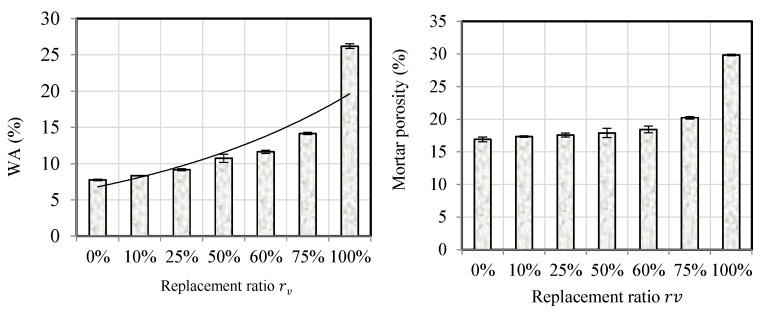
Water absorption coefficient and porosity evolution of mortars versus replacement ratio of NS by CR.

**Figure 9:**
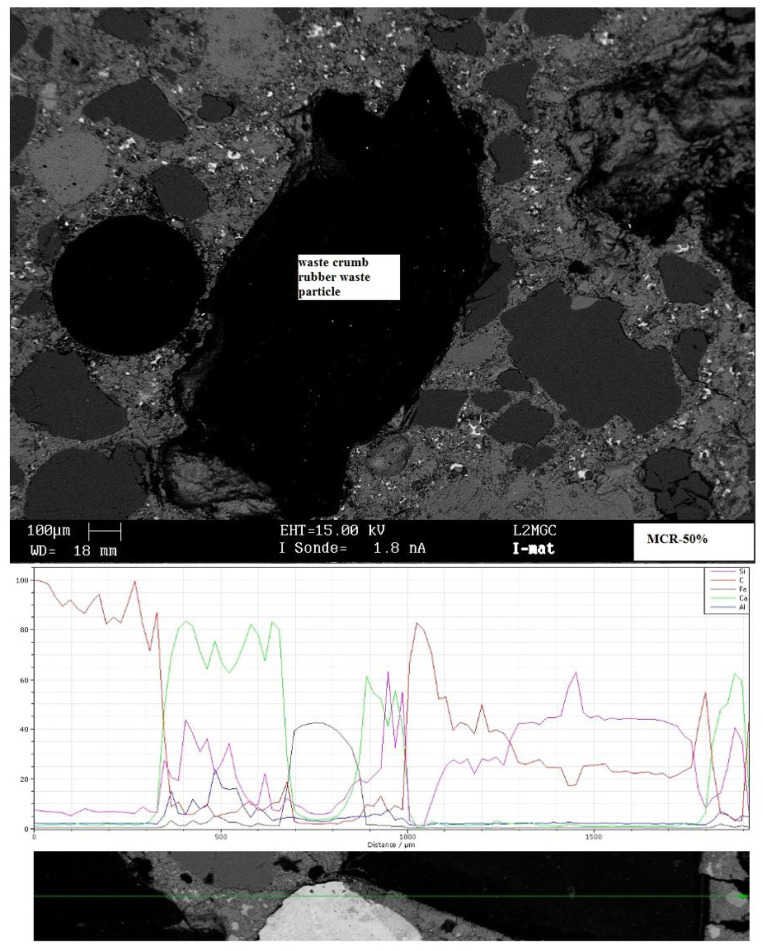
Scanned Electron Microscopy images indicating the quality of adhesion between cement binder and rubber particles.

**Figure 10:**
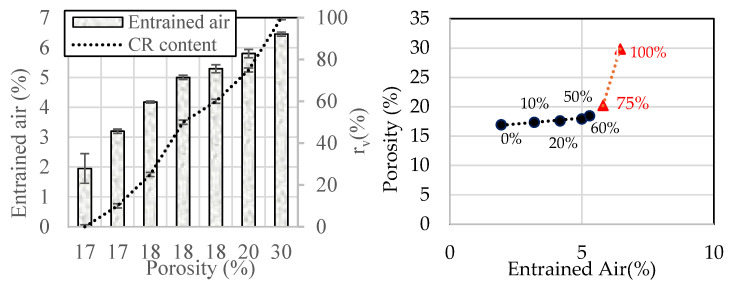
Effects of CR incorporation on the porosity and air content of the mortar mixes.

**Figure 11:**
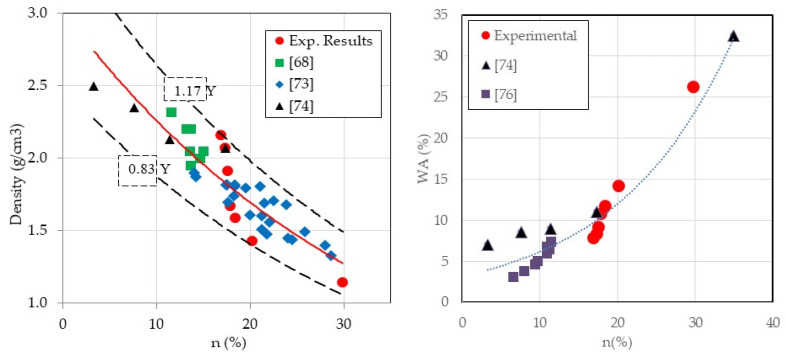
Evolution of the mortar density and water absorption coefficient versus its porosity [68,73,74,76].

**Figure 12:**
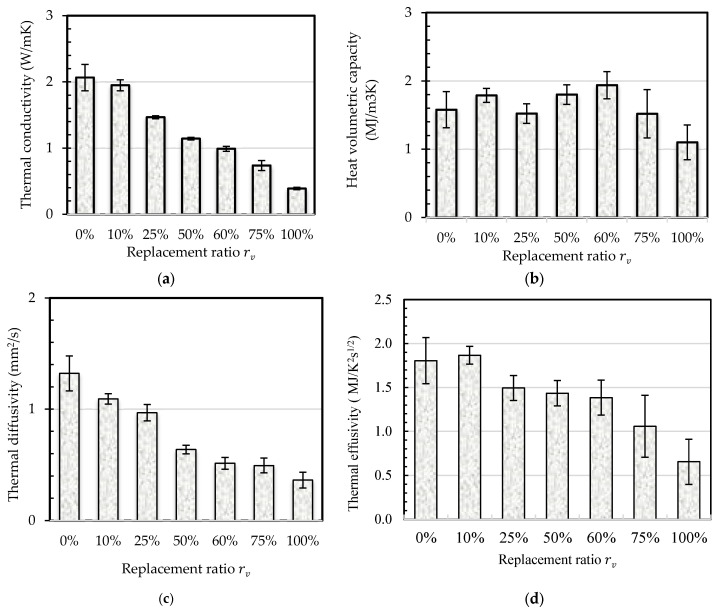
Thermal properties of the mortars as a function of the CR content (%).

However, it should be noted that the mortars developed do not comply with the French Environmental Regulation 2020 “RE 2020” [78]. The latter suggests minimum thermal resistances R ranging from 2.1 to 3.2 W/m^2^ for renovated wall and floor insulation solutions, depending on the climatic zone. Moreover, RE 2020 typically suggests an average insulation thickness of 15 to 20 cm for walls, 20 to 25 cm for ceilings, and 10 to 20 cm for floors. For a thickness of 20 cm, the developed mortars fail to meet the RE 2020 requirements, despite showing a significant increase in thermal resistance of over 50% as seen on the left side of Figure 13. This can be expressed by the following equation:

$$R=\frac{e\left(m\right)}{\lambda \left(W/\mathrm{mK}\right)}=0.088e^{0.016r_{v}\left(\%\right)}\;\mathrm{with}\;R^{2}=0.965.$$

Several authors have attempted to correlate thermal conductivity with density [79,80,81]. In order to avoid the effects of control mortar-related parameters affecting these two properties and to study only the impact of incorporating crumbled rubber tire particles, it is proposed in this study to establish a relationship between the normalized thermal conductivity $\left(\lambda /\lambda_{o}\right)$ and the normalized density $\left(\rho /\rho_{o}\right)$. An increase in normalized thermal conductivity is evident as the normalized density increases on the right side of Figure 13. The correlation between these two parameters is accurately depicted by the following equation:

$$\frac{\lambda }{\lambda_{o}}=0.048e^{2.978\left(\frac{\rho }{\rho_{o}}\right)}\;\mathrm{with}\;R^{2}=0.88$$

$\lambda_{o}$ and $\rho_{o}$ represent the thermal conductivity and density of the control mortar.

The decrease in thermal conductivity with higher CR content indicates improved insulation properties, which is beneficial for energy efficiency in buildings. However, the reduction in thermal diffusivity and effusivity suggests that the mortar’s ability to store and release heat is diminished. This trade-off between insulation and thermal inertia must be considered when designing mortars for specific applications, particularly in climates with significant temperature fluctuations.

c. Drying/Shrinkage

Given the influence of crumb rubber on porosity and water absorption, it is important to analyze its impact on drying and shrinkage. Drying and shrinkage are important causes of damage in concrete structures, especially when contraction is prevented, which induces tensile stresses. It is known that shrinkage increases with the volume fraction of paste in concrete and reduces with relative humidity [66]. In this study, we investigated the effect of fine crumb rubber on these phenomena when humidity, water-to-cement ratio, and water content were fixed.

**Figure 14:**
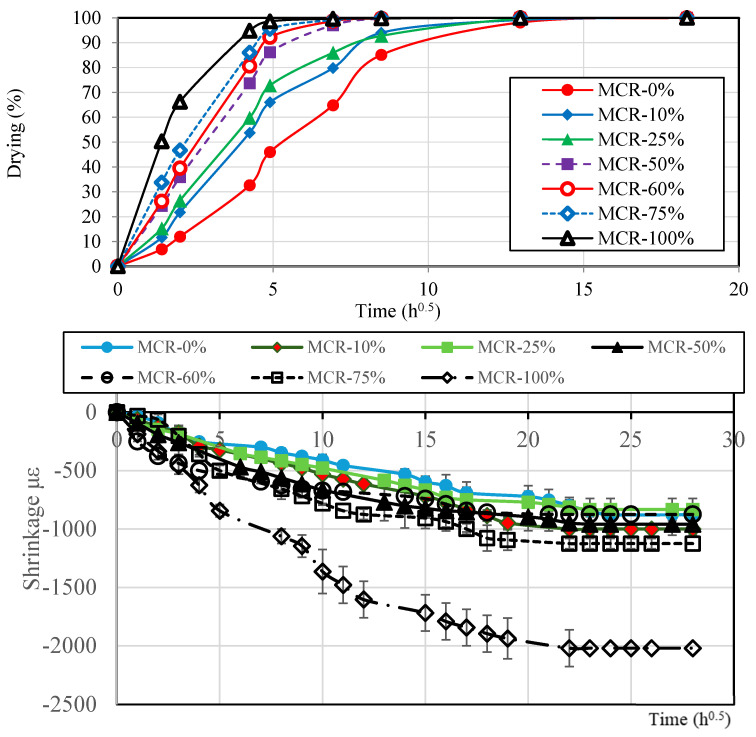
Drying versus time $\sqrt{\mathrm{hours}}$ and shrinkage time (days) for various mixes.

The results in terms of drying shrinkage of mortar with crumb rubber compared to plain mortar are shown in Figure 14. These results reveal that crumb rubber had a significant effect on drying; while MCR-0% took about 15 h to fully dry, MCR-100% dried within 5 h, which indicates that rubberized mortar dries 3 times faster. This can be explained by the abundance of pore spaces and high water absorption in mortar with high CR replacement ratios. The results also show that shrinkage increased significantly with the crumb rubber content beyond 60%. This can be explained by the weaker structural performance of rubber particles compared to sand particles; the formers being much more flexible offer low confinement for mortar which leads to higher shrinkage [65]. It is known that shrinkage occurs during cement hydration as the hardening phases continue to cure [71]. Shrinkage strongly impacts the overall strength of cement mortar as it is associated with microcracks that concentrate stresses and may lead to failure. It can be noticed that the shrinkage of all the mixes is quite similar for $r_{v}\leq 60\%$; the obtained values at 28 days vary between −875 με to −1000 με.

The increased drying rate and shrinkage with higher CR content highlight the need for careful management of these properties. While faster drying can be advantageous for reducing construction time, increased shrinkage can lead to cracking and compromised structural integrity. The findings suggest that CR content that balances these effects, ensuring that the mortar retains sufficient strength and durability over time should be $r_{v}\leq 75\%$.

d. Compressive strength

**Figure 15:**
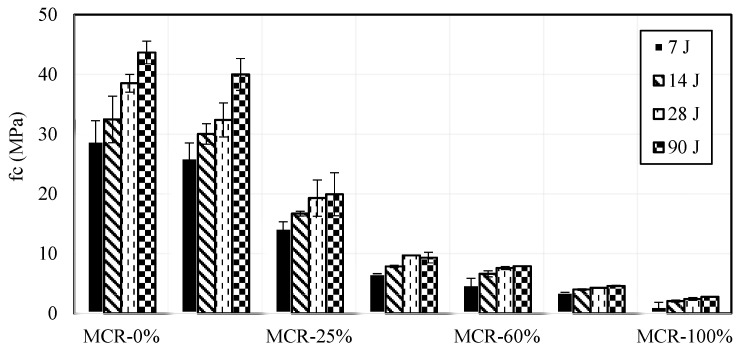
Effect of crumb rubber content on compressive strength.

Mechanical characteristics of mortar were assessed at intervals of 7-, 14-, 28-, and 90-days post-curing. Figure 15 displays the experimental results for compressive strength. The compressive strength consistently increases over time during the curing process, regardless of the replacement ratio. At a 10% CR content, the compressive strength rose from 28.85 MPa at 7 days to 43.68 MPa at 90 days. At a constant age, the compressive strength decreases as the CR replacement ratio increases, as indicated by the figure. Specimens aged 28 days showed strengths of 38.52 MPa, 32.38 MPa, 19.28 MPa, 9.7 MPa, 7.7 MPa, 4.27 MPa, and 2.41 MPa at CR contents of 0%, 10%, 25%, 50%, 60%, 75%, and 100%, respectively. There is a notable decrease in resistance after surpassing 25%.

The NF EN 1504-3 standard defines four classes of repair products according to the performance of mortars: structural repair mortars (e.g., Class R4 for $f_{c}\geq 45\;\mathrm{MPa}$ and Class R3 for ($f_{c}\geq 25\;\mathrm{MPa}$) and non-structural repair mortars (Class R2 for $f_{c}\geq 15\;\mathrm{MPa}$ and Class R1 for $f_{c}\geq 10\;\mathrm{MPa}$). It can be observed that up to 50% of CR content, the proposed mortars are within the range prescribed by the standard but can be used solely as non-structural repair products for civil engineering buildings.

**Figure 16:**
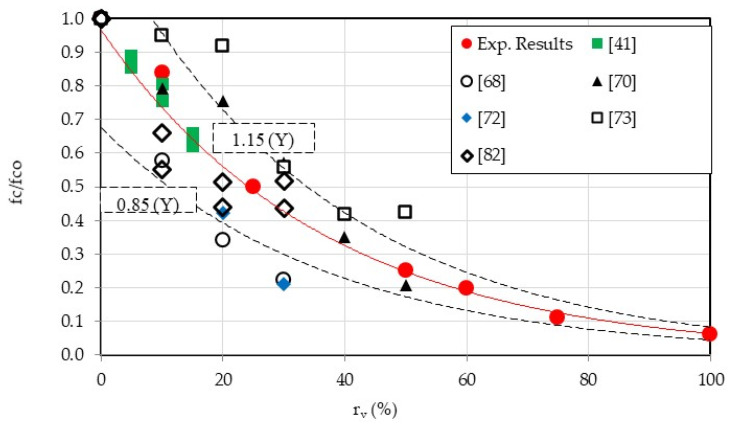
Effect of crumb rubber content on the normalized compressive strength—comparison with published data [41,68,70,72,73,82].

EN 206-1 specifies that lightweight concretes used for structural purposes should have a minimum compressive strength class of LC8/9 at 28 days, with density classes ranging from D1.0 to D2.0. Mortars containing up to 50% CR meet the requirements of EN 206-1. Figure 16 compares the outcomes of the current study, which encompass a broad spectrum of CR contents, with previous research. In general, our findings align with the mean of existing data [41,68,70,72,73,82].

Based on the published data and the current results, an empirical expression of compressive strength at a replacement ratio CR was obtained:

$$\frac{f_{c}}{f_{co}}=0.97e^{-0.03r_{v}}\;\mathrm{with}\;R^{2}=0.85$$

where $f_{co}$ is the compressive strength of control mortar, $r_{v}$ is the ratio of rubber/sand replacement (ranging from 0 to 100%).

**Figure 17:**
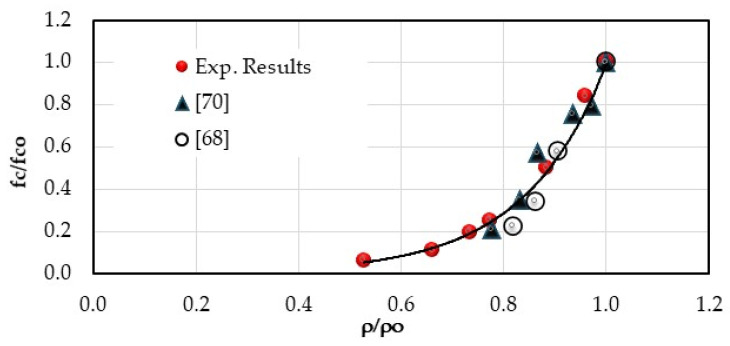
Evolution of compressive strength of rubberized mortars [68,70].

Moreover, it appears that a relationship can be established between the compressive strength and the density of mortars incorporating CR (Figure 17):

$$\frac{f_{c}}{f_{co}}=0.002e^{6.21\left(\frac{\rho }{\rho_{o}}\right)}\;\mathrm{with}\;R^{2}=0.97$$

The decrease in compressive strength with increasing CR content is expected due to the lower stiffness and strength of rubber compared to natural aggregates. However, mortars with up to 50% CR content still meet the requirements for non-structural repair applications, indicating their potential for use in less demanding structural elements. The established relationships between compressive strength and CR content or density provide a useful guideline for designing mortars with specific strength requirements.

e. Flexure/Tensile strength

**Figure 18:**
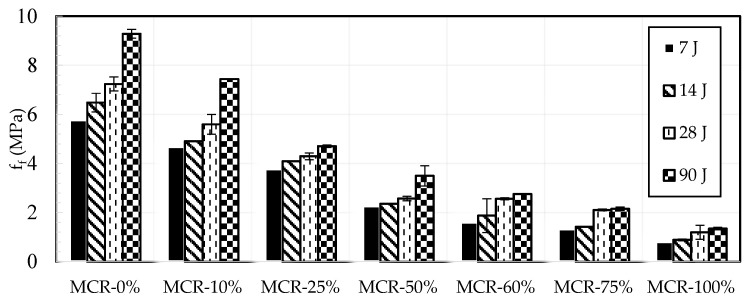
Effect of crumb rubber content on flexural strength during the curing.

The experimental results in terms of flexural strength are presented in Figure 18. As for compressive strength, it can be seen that it increases with time irrespective of the replacement ratio. For example, at 25% CR content, flexural strength increased from 3.8 MPa at 7 days to 4.71 MPa at 90 days. However, the figure shows that at a fixed age, flexural strength reduced with the CR replacement ratio. For example, specimens of age 90 days have strengths of 9.3 MPa, 7.45 MPa, 4.71 MPa, 3.5 MPa, 2.75 MPa, 2.15 MPa, and 1.35 MPa when the CR content is 0%, 10%, 25%, 50%, 60%, 75%, and 100%, respectively. In coherence with the compressive strength tests, the results show a significant drop in resistance, as can be seen beyond 25%.

**Figure 19:**
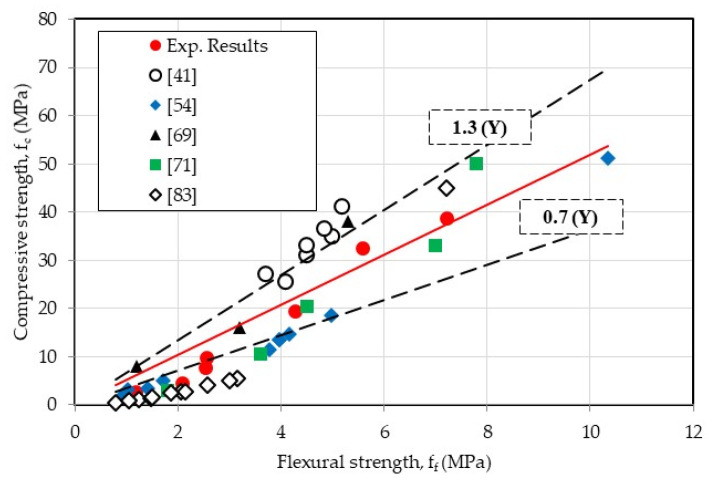
Correlation between flexural and compression strength [41,54,69,71,83].

Figure 19 compares the results of the present work with previous studies in terms of compressive strength versus flexural strength. Overall, our results are comparable with the average of published data despite the discrepancy that can be seen around the average [41,54,69,71,83]. It should be remembered that our experimental campaign covered a wider range of CR content ($0\leq r_{v}\left(\%\right)\leq 100)$. Based on the published data and the current results, an expression between flexural and compressive strengths of the different mortars is established:

$$f_{c}=5.36f_{f}\;\mathrm{with}\;R^{2}=0.77$$

**Figure 20:**
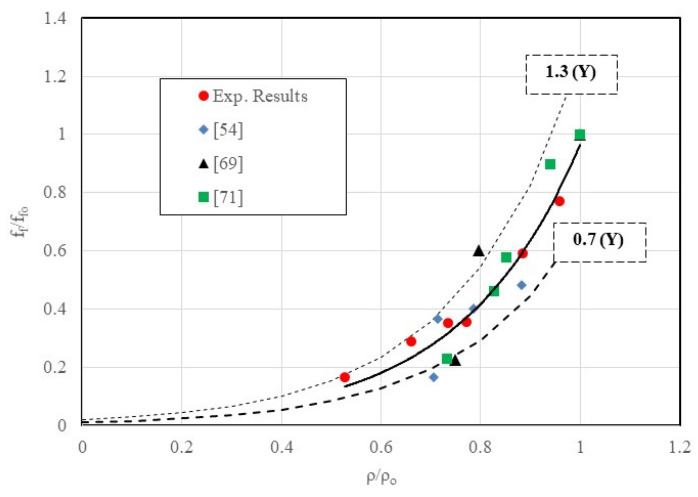
Evolution of the normalized flexure strength versus normalized density of rubberized mortars [54,69,71].

To assess the effect of incorporating tire crumb rubber particles in mortars, normalized flexural strength is plotted against normalized density, and a relationship between these two properties is established (Figure 20). It can be outlined that the decrease in the relative density due to the presence of CR particles leads to a decrease in the relative flexure strength. It can be outlined that the decrease in the relative density due to the presence of CR particles leads to a decrease in the relative flexure strength:

$$\frac{f_{f}}{f_{fo}}=0.015e^{4.19\left(\frac{\rho }{\rho_{o}}\right)}\;\mathrm{with}\;R^{2}=0.85$$

where $f_{fo}$ is the flexure strength of the control mortar.

**Figure 21:**
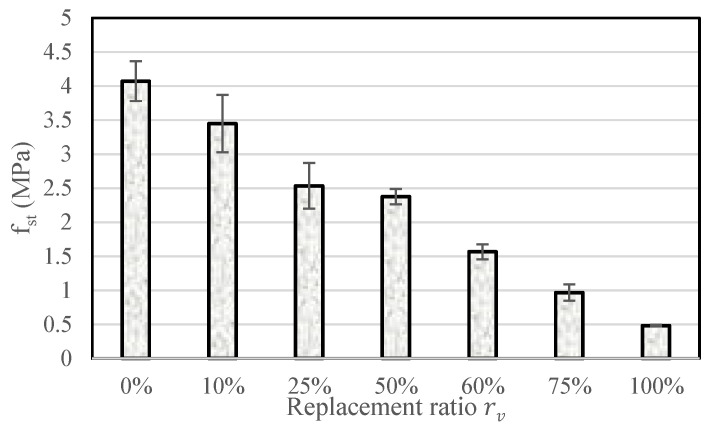
Effect of crumb rubber content on splitting tensile strength, f_st_.

While the NF EN 1504-3 standard for repair mortars does not specify a tensile strength requirement, direct tensile tests were carried out on cylindrical specimens measuring 11 cm in diameter and 22 cm in length. The results are shown in Figure 21 with a significant decrease in tensile strength observed for CR content above 25% and an overall mechanically more ductile behavior for $r_{v}\geq 75\%$. However, the tensile strength can be considered satisfactory up to 60% as it is higher than 1.5 MPa.

Similar to compressive strength, flexural and tensile strengths decrease with higher CR content. The significant drop in strength beyond 25% CR suggests a threshold for maintaining adequate mechanical performance. The correlation between flexural and compressive strengths offers a practical way to estimate the mortar’s flexural capacity based on its compressive strength, aiding in the design and application of CR-containing mortars.

f. Fracture energy

**Figure 22:**
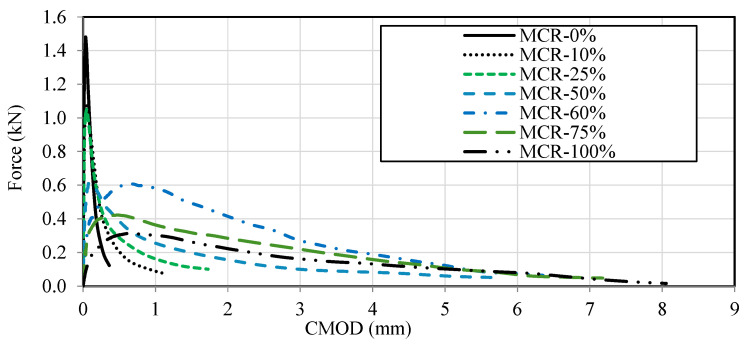
Load–displacement response of the structure used to predict the fracture energy.

Figure 22 shows the load-CMOD curves obtained for various CR replacement ratios. Similar results were obtained in terms of force versus deflection. These results underline a more resilient behavior and greater resistance to crack propagation as the incorporation rate of crumb rubber tires particles increases. This is particularly obvious at ratios in excess of 50%.

The fracture energy is calculated according to RILEM [58] formula:

$$G_{f}=\frac{W+\left(m_{b}+2m_{l}\right)g\delta_{0}}{A_{lig}}$$

where *W* is the area below the load–deflection curve, $m_{b}$ is the mass of the beam portion between the support points, $m_{l}$ is the mass of any support arrangement excluding the machine, g is the gravitational acceleration constant, $\delta_{0}$ is the deflection upon failure, and $A_{lig}$ is the fracture zone area.

**Figure 23:**
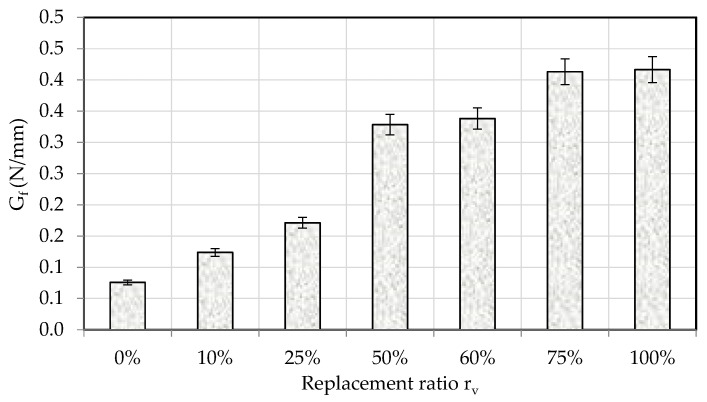
Fracture energy at various CR contents.

The addition of crumb rubber (CR) to cement-based mortars significantly affects their fracture energy, an essential factor for assessing the material’s resistance to crack propagation. Figure 23 depicts the relationship between fracture energy and the CR replacement ratio. The data indicates a distinct trend: an increase in CR content correlates with a rise in the fracture energy of the mortar, particularly for $r_{v}\geq 50\%$. This observation is consistent with previous studies that have reported similar effects when integrating CR into cementitious materials.

The rise in fracture energy with elevated CR content is due to the development of a more extensive microcrack network in the mortar matrix. Crumb rubber particles, due to their softer and more flexible nature compared to traditional aggregates, promote the formation of microcracks. When the material experiences stress, microcracks tend to merge and spread, thereby absorbing energy during this process. This mechanism of energy dissipation contributes to improved fracture resistance. Numerous studies in the literature have documented analogous findings concerning the impact of CR on fracture energy. Studies have consistently demonstrated that the incorporation of CR in cement-based materials enhances fracture energy, primarily attributable to the mechanisms outlined above. The improved capacity of CR-modified mortars to absorb and dissipate energy during fracture renders them especially appropriate for applications requiring resistance to crack propagation. The enhanced fracture energy of mortars containing CR has significant implications for their application in construction. Mortars exhibiting elevated fracture energy demonstrate superior resistance to cracking under stress, thereby improving the durability and longevity of structures. This characteristic is significant in applications like the repair and rehabilitation of concrete structures, where the material’s resistance to cracking is crucial for preserving structural integrity.

g. Elastic modulus

**Figure 24:**
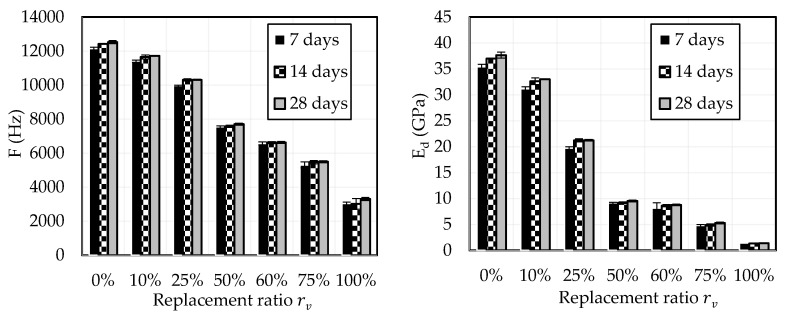
Resonance frequency and dynamic modulus of elasticity (Ed) per NF EN ISO 12680-1, based on SN to CR replacement ratio in mortars, for various curing times.

The left-hand side (LHS) of Figure 24 illustrates that the resonance frequency of the mortar decreases as the Crumb Rubber (CR) content increases, for any given curing time. Notably, at 28 days of curing, the frequency decreases from 12,526 Hz to 3305 Hz as CR content increases from 0% to 100%. This trend indicates an enhanced damping ability in mortars with higher CR content, as the material more effectively absorbs and dissipates vibrational energy.

The right-hand side (RHS) of Figure 24 shows the variation in the dynamic modulus of elasticity (Ed) with respect to CR replacement and curing time. The modulus exhibits a similar trend to the resonance frequency, decreasing with increasing CR content for a given curing age. This suggests that higher CR content in mortars leads to increased creep ability, as the material becomes more flexible and less stiff.

Both the resonance frequency and the dynamic modulus of elasticity exhibit slight increases with curing time, although this effect is less pronounced for CR replacement ratio $r_{v}\geq 50\%$.

According to the NF EN 1504-3 standard, structural repair mortars must have an elastic modulus exceeding 20 GPa for class R4 and 15 GPa for class R3. There are no specific elastic modulus requirements for non-structural repair classes. Based on these criteria, mortars with up to 25% CR replacement can be employed as structural repair products in civil engineering applications. Conversely, mortars with higher CR replacement ratios are more suited for non-structural applications due to their lower elastic modulus.

**Figure 25:**
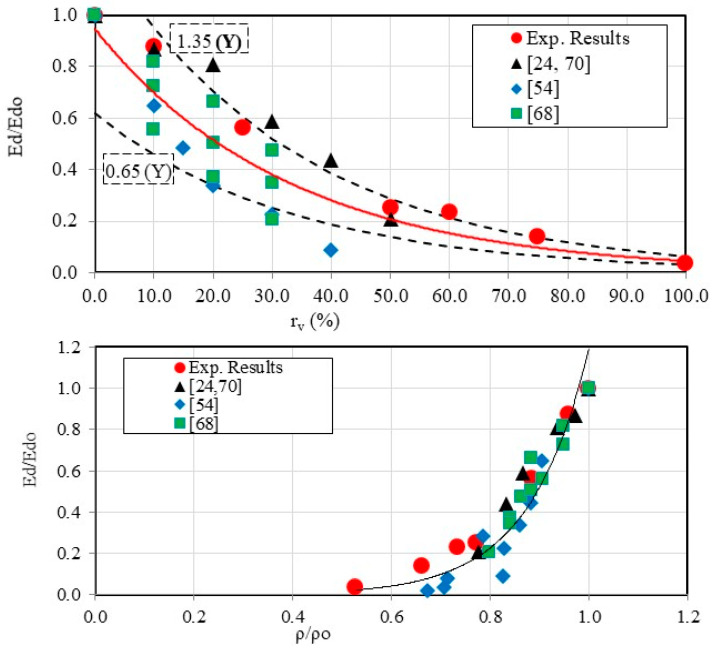
Dynamic modulus of the different mortars [24,54,68,70].

The evolution of the dynamic modulus $E_{d}$ against the density $\rho_{ap}$ derived from our experimental results and corroborated by published data [24,54,68,70] is illustrated in Figure 25. The figure validates the consistency between our findings and existing literature while extending the analysis to a broader range of crumb rubber replacement ratios (for instance $0\leq r_{v}\left(\%\right)\leq 100$ while $r_{v}\left(\%\right)\leq 50$ in the literature). Furthermore, the relationship between the elastic modulus and the crumb rubber content exhibits a trend similar to that observed for compressive strength, described by the equation:

$$\frac{E_{d}}{E_{do}}=0.95e^{-0.03r_{v}}\;\mathrm{with}\;R^{2}=0.79$$

where $E_{d0}$ is the elastic modulus of plain mortar and $r_{v}$ is the ratio of rubber/sand replacement ratio (%).

It is also interesting to highlight that a relationship has been established linking the normalized modulus to the normalized density, demonstrating that as the density decreases, the resistance to elastic deformation also decreases for mortars incorporating tire crumb rubber particles:

$$\frac{E_{d}}{E_{do}}=0.0003e^{8.29\left(\frac{\rho }{\rho_{o}}\right)}\;\mathrm{with}\;R^{2}=0.82$$

The decrease in the dynamic modulus of elasticity with increasing CR content reflects the mortar’s reduced stiffness. While this may limit its use in structural applications requiring high stiffness, it also indicates improved damping properties, which can be advantageous for reducing vibrations and noise. The relationship between the elastic modulus and CR content provides valuable insights for designing mortars with specific stiffness and damping characteristics.

### 5.3. Pull Out

This section discusses the results of pull-out tests conducted to evaluate the bond strength between concrete substrates and mortars containing varying amounts of crumb rubber (CR) as a replacement for natural sand (NS). The goal is to assess the adhesion properties of these mortars, which is crucial for their application in the repair and rehabilitation of concrete structures.

**Figure 26:**
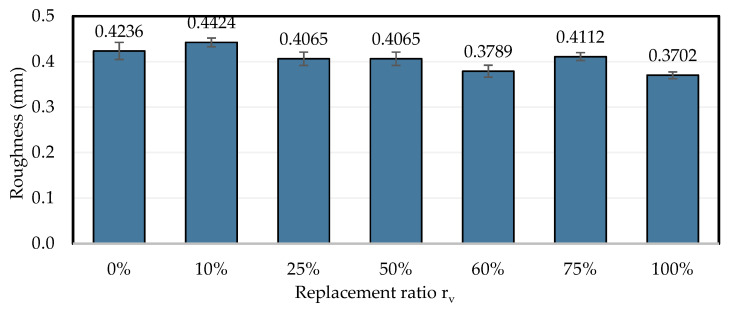
The roughness of the concrete surface before repairing.

The roughness values obtained for different concrete slabs where each rubberized mortar formula was applied are shown in Figure 26. The roughness values obtained are very close, ranging between 0.44 mm and 0.37 mm. This indicates that the concrete surfaces were prepared consistently across different samples. Moreover, consistent roughness ensures that variations in bond strength are primarily due to the properties of the mortar rather than differences in surface preparation.

**Figure 27:**
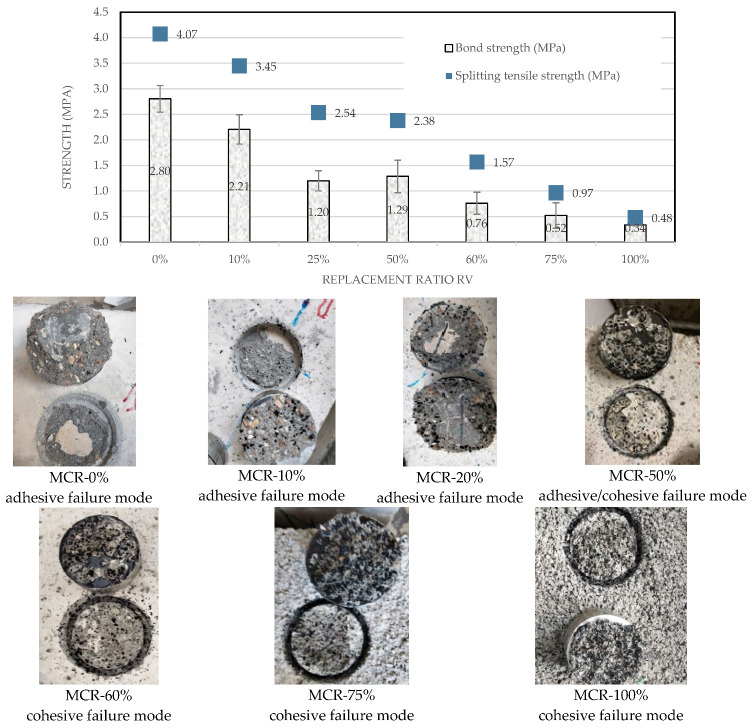
Pull out tests results.

The bond strength decreases when the mortar contains more CR ratio (Figure 27). However, adhesion remains of good quality and no decohesion has been observed. The decrease in bond strength with increasing CR content is expected due to the lower stiffness and strength of rubber compared to natural sand. Indeed, the consistent roughness of the concrete surfaces ensures that the bond strength variations are attributable to the mortar composition rather than surface preparation differences. This analysis supports the potential use of CR-containing mortars for repair and rehabilitation, aligning with the study’s objectives of reducing environmental impact and promoting the recycling of ELTs.

The obtained bond strengths values are slightly lower than the tensile strength values of the rubberized mortars (Figure 27). The higher tensile strength compared to bond strength reflects the inherent differences between the bulk properties of the mortar and the interfacial properties between the mortar and the substrate.

The NF EN 1504-3 standard prescribes bond strengths ≥2 MPa for Class R4; ≥1.5 MPa for Class R3, ≥0.8 MPa for class R2 and no specific requirement for Class R1. It can be observed that mortars with $25\leq r_{v}\leq 60\%$ meet the bond strength requirement for Class R2 (≥0.8 MPa), making them suitable for non-structural repair applications.

The failure mode was adhesive for $r_{v}\leq 50\%$, cohesive for $r_{v}=100\%$ and mixed for $60\%\leq r_{v}\leq 75\%$. The absence of decohesion suggests that the mortar adheres well to the concrete substrate, even with higher CR content leading to a failure within the mortar layer near the interface rather than at the interface itself. This indicates that the mortar maintains a strong bond with the concrete substrate, which is crucial for repair applications.

Figure 27 provides valuable insights into the bond strength of repair mortars with varying CR content. The decrease in bond strength with increasing CR content is expected, but the mortars still meet the requirements for non-structural repair applications. The absence of decohesion and the comparison with tensile strength values highlight the potential of these mortars for sustainable and effective concrete repair solutions.

### 5.4. Fire Resistance of Cured Mortars

**Figure 28:**
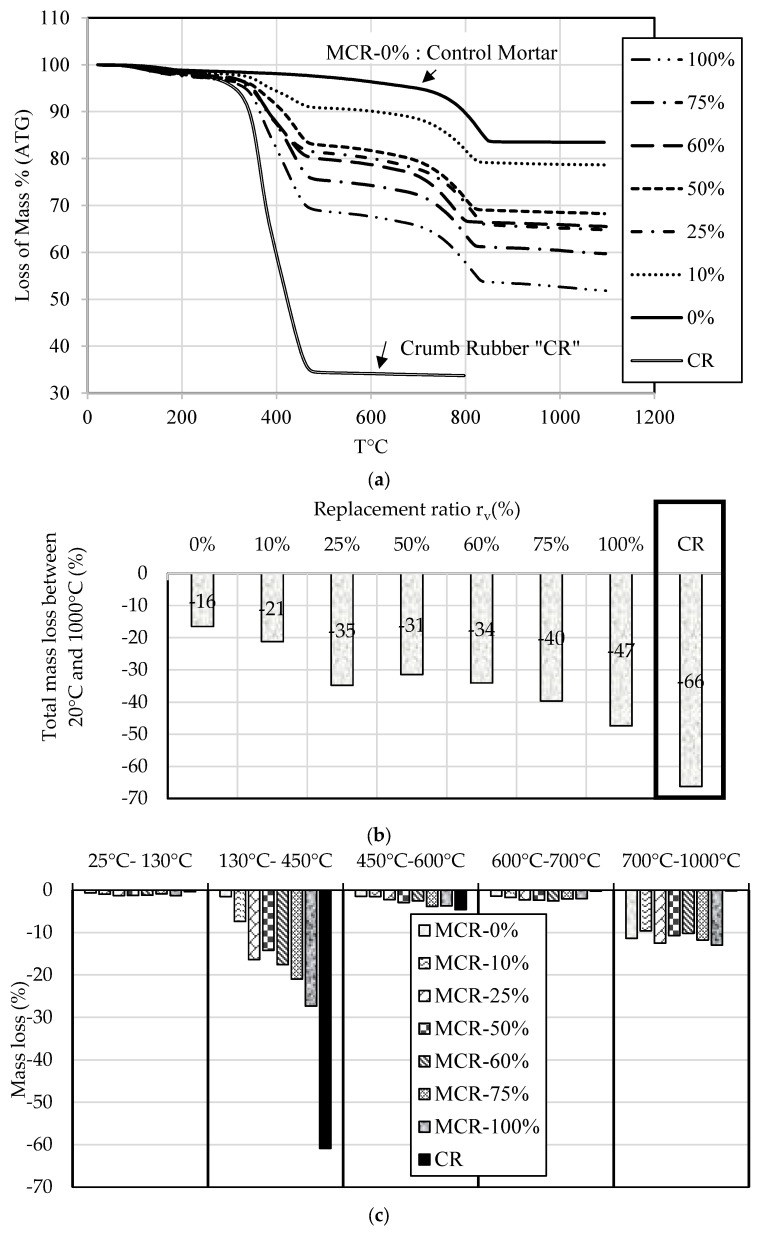
(**a**) TGA curves of CR aggregates and the different mortar mixes. (**b**) Total mass loss of the mortars (%) as a function of $r_{v}\left(\%\right)$. (**c**) Mass loss (%) in the different range of temperatures.

As shown in Figure 28a, mass losses increased with the increase in temperature and crumb rubber content. In other words, mortar embedding crumb rubber lost more weight than plain mortar when temperature increased (Figure 28b). This is mainly explained by the increase in the CR content characterized by the highest value of total mass loss.

The thermal gravimetric results reported in Figure 28c indicate that CR mass loss was about 61% between 130 °C and 450 °C. This increase is explained by the pyrolysis of polyisoprene and the decomposition of butadiene from vulcanized rubber.

For mortars, the increase in mass loss with temperature resulting from phase change in the cement paste is well described in the literature [84,85]. Based on this study, it can be concluded that:

- Between room temperature and 150 °C, the variation of mass is essentially due to the release of volatiles (water and organic compounds).
- Between 450 °C and 600 °C the mass loss is mainly attributed to the decomposition of the portlandite ($\mathrm{Ca}{\left(\mathrm{OH}\right)}_{2}\to \mathrm{CaO}+H_{2}O$) and the end of the decomposition of the crumb rubber particles.
- Between 600 °C and 700 °C the mass loss is due to the dehydration of the CSH gel.
- Beyond 700 °C to 1000 °C, the loss of mass is attributed to the decomposition of calcite (${\mathrm{CaCO}}_{3}\to \mathrm{CaO}+{\mathrm{CO}}_{2}$) and the end of CSH decomposition.

In light of the analysis of thermal gravimetric results, mortar specimens at 28 days of age, with CR to NS replacement ratios of 0%, 10% and 25%, were placed in an oven for 24 h at temperatures of 300 °C (where the effect of CR is predominant: decomposition and pyrolysis), 450 °C (decomposition of CR and portlandite) and 600 °C (decomposition of CR, portlandite and dehydration of CSH). After heat treatment, the specimens were cooled at ambient conditions and weighed to evaluate their mass losses.

**Figure 29:**
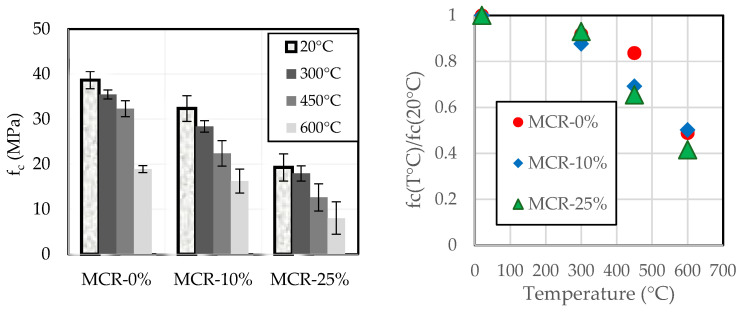
Effect of temperature on the strength of mortars embedding CR.

The variation of compressive strength in mortar incorporating CR due to exposure to high temperature are shown in Figure 29. The curves show that the compressive strength decreases with the increase in the temperature of exposure. However, this decrease is independent of CR content, as shown in the RHS of Figure 29, where the compressive strength of the mortar after exposure at a specific temperature is normalized by the strength obtained at room temperature.

The increased mass loss observed with higher crumb rubber (CR) content during thermal exposure highlights the importance of assessing fire resistance in CR-containing mortars. Despite this mass loss, the structural integrity and resistance of these mortars do not decrease after fire exposure, indicating robust performance under thermal stress. This finding suggests that CR-containing mortars with a rubber volume replacement ratio $r_{v}\leq 25\%$ can maintain their structural properties without additional fire protection measures, provided the mass loss remains within acceptable limits. Furthermore, the total mass loss results (as depicted in Figure 28b,c) suggest that similar conclusions should be drawn for mortars with $r_{v}$ up to 60%. The results underscore the potential of CR-containing mortars (when $r_{v}\leq 60\%$) to offer both durability and fire resistance, making them a viable option for applications where thermal stability is crucial.

## 6. Conclusions

This research aims to create new mortars for repair and rehabilitation that minimize ground overloading, decrease energy consumption in buildings, and enhance the recycling capabilities of ELTs as a “recovery material”. Furthermore, utilizing scrap tires will decrease the reliance on natural sand, a resource that is the second most utilized globally after water and is at risk of running low.

Seven mixtures were created and tested, consisting of control mortars and mortars with varying proportions of crumb rubber particles (0%, 10%, 25%, 50%, 60%, 75%, and 100%). All experimental tests were conducted in accordance with applicable standards. The objective was to analyze the behavior of crumb rubber particles, the behavior of various mixtures in fresh and hardened states, and the efficacy of mortars with the highest percentage of crumb rubber particles as repair/rehabilitation materials. The results were compared with those in the published literature to establish relationships between mortar properties and crumb rubber particle content. The chosen mortars were exposed to high temperatures to simulate the effects of fire on compressive strength. “The results indicate that mortars with up to 50% CR content meet the standards for non-structural repair applications, with a significant improvement in thermal insulation properties. Specifically, the thermal conductivity decreased by up to 82% with 100% CR content, and the compressive strength remained within acceptable limits for non-structural repairs. These findings highlight the potential of using recycled tire-derived aggregates in lightweight mortars for sustainable and effective concrete repairs”.

Below are the most important detailed findings:

- Crumb rubbers obtained from EOL tires can be used with confidence as aggregates in mortars. Leaching tests revealed low levels of leached pollutants, confirming that CR particles can be considered safe for both health and the environment, particularly when embedded in a cementitious matrix.
- The air content of early-age mortar paste increases linearly with the CR replacement ratio. Entrapped air bubbles have a significant effect on the hardened behavior since they can concentrate stresses or facilitate the infiltration of damaging elements.
- The increase in the CR content reduces the setting time and the workability of mortars. However, mixes with $r_{v}\leq 60\%$ can still be used for repair work as they meet recommended standards.
- The apparent density decreases as the CR fraction increases. According to standards, mortars with $r_{v}\geq 25\%$ are classified as lightweight mortars. Moreover, an increase in porosity, mainly attributed to the higher occluded air content associated with higher CR rates, is observed particularly for $r_{v}>50\%$.
- Since the number and volume of pore spaces and the surface area of cement–rubber interfaces vary with CR content, water absorption increases accordingly. In this study, it was established that both the normalized water absorption coefficient and the bulk density vary in the same way with the CR replacement ratio: $\frac{\rho_{ap}}{\rho_{ap,o}}=\frac{WA}{WA_{o}}=e^{-0.01r_{v}}\;.$
- Similarly, drying shrinkage increases with time and CR content due to the corresponding increase in the number and volume of pore spaces and cement–rubber interfaces. However, it is established that this increase is more significant for mixes with $r_{v}>60\%$.
- The mechanical properties decrease as the CR content increases. This was verified in terms of compressive, flexural and tensile strengths as well as the elastic modulus. Nevertheless, mortars with $25\leq r_{v}\left(\%\right)\leq 50$ can be used as lightweight mortars according to standard recommendations. Up to 50% of CR content, the proposed mortars fall within the range prescribed by the standard as non-structural repair products for civil engineering buildings.
- A similar relationship links the normalized compressive strength and normalized modulus of elasticity to rubber content $\frac{f_{c}}{f_{co}}=\frac{E}{E_{o}}=e^{-0.03r_{v}}$. This relationship was established on the basis of our new experimental results and those reported in the literature. However, it should be emphasized that all normalized mechanical properties are highly dependent on the normalized density. Expressions relating to these properties have been established to isolate the effect of rubber incorporation.
- Incorporating up to 50% of waste tires improves comfort and thermal resistance without affecting thermal inertia. Experimental data indicate that thermal conductivity significantly decreases with respect to CR. However, the changes in volumetric heat capacity with CR are not as large, which explains the little changes observed in terms of thermal effusivity.
- The fracture energy increases with the increase in CR content. The increase in the density of microcrack networks and aggregate–cement interfaces weakens the material embedding soft crumb rubber. These defects coalesce and propagate, which results in an increase in energy dissipation with higher CR replacement ratios.
- Pull-out test results show that the bond strength decreases with increasing CR content. However, the obtained bond strengths conform to standards for non-structural repair applications when $25\leq r_{v}\leq 60\%$.
- Mass losses due to heat treatment increase with higher crumb content and/or temperature. However, the variations remain below 12% in the temperature range considered (20 to 600 °C).

In summary, mortars embedding CR can be used to lighten structures given their low density. When applied to the repair/rehabilitation structures, lightweight mortar provides additional strength without hindering the portability. The mortars developed with $25\leq r_{v}\leq 50\%$ can be classified as lightweight mortars with improved thermal comfort according to standards and be used as non-structural repair products.

## Figures and Tables

**Table 1:** ELT generated in various countries (in metric kilotons) and recovered for various purposes.

Country	Total ELT Generated	Energy	Material	Civil/Backfill	Other	Total ELT Recovered	ELT Recovered (%)
China (2018)	14,545	0	5650	0	8895	5650	39
United States (2017)	3700	1442	1227	326	706	2995	81
Europe (2017)	3425.5	1180	1855.5	105.5	283.5	3141	92
India (2015)	2749.8	600	2094.8	0	55	2694.8	98
Japan (2017)	849	619.5	160.5	1	68	781	92
Russia 2017)	800	6	154	0	640	160	20
Indonesia (2017)	684.4	376.4	136.9	0	171.1	513.3	75
Brazil (2017)	587.9	206.1	379.1	0	2.7	585.2	100
Thailand (2012)	515	75.4	202.3	0	237.3	277.7	54
Mexico (2017)	467.5	67.1	27.9	0	372.5	95	20
South Korea (2017)	319.4	160	120.9	0	38.5	280.9	88
South Africa (2015)	204	9.4	41.5	0	153	50.9	25
Argentina (2018)	150	0	9.6	0	140.4	9.6	6
Nigeria (2017)	113	2.8	2.8	0	107.3	5.6	5

**Table 2:** ELTs generated and recovered in Australia in tons.

		Passenger/Motorbike	Truck/Bus	Off-the-Road	Total
2009–2010					
	Consumption of new tires	168,901	156,095	173,382	498,377
	Generation of waste tires	105,581	117,391	164,775	387,747
	Recovery/Reuse of waste tires	79,060	43,476	9568.416	132,104
	Waste tires recovery rate	0.75	0.37	0.06	0.34
2013–2014					
	Consumption of new tires	154,518	183,682	198,887	537,087
	Generation of waste tires	122,686	127,369	158,276	408,331
	Recovery/Reuse of waste tires	88,335	53,430	12,299	154,064
	Waste tires recovery rate	0.72	0.42	0.08	0.38
2018–2019					
	Consumption of new tires	223,000	195,000	127,000	545,000
	Generation of waste tires	188,000	156,000	119,000	463,000
	Recovery/Reuse of waste tires	167,320	138,840	13,090	319,250
	Waste tires recovery rate	0.89	0.89	0.11	0.69
2019–2020					
	Consumption of new tires	226,000	197,000	128,000	551,000
	Generation of waste tires	185,000	152,000	113,000	450,000
	Recovery/Reuse of waste tires	164,650	136,800	15,820	317,270
	Waste tires recovery rate	0.89	0.90	0.14	0.71
2021–2022					
	Consumption of new tires	227,600	194,400	141,000	563,000
	Generation of waste tires	187,600	157,800	113,600	459,000
	Recovery/Reuse of waste tires	169,800	146,300	14,200	330,300
	Waste tires recovery rate	0.91	0.93	0.13	0.72

**Table 3:** Density and water absorption of NS and CR.

Materials	Size (mm)	ρ_rd_ (g/cm^3^)	WA_24h_ (%)	$R_{s}$
Natural sand (NS)	0.063–5	2.58 ± 0.02	0.98 ± 0.1	1.2
Crumb rubber (CR)	0.5–5	0.91 ± 0.01	0.2 ± 0.1	

**Table 4:** Concentrations of inorganic species leached in contact with water.

Element	Concentration Measured mg/L	Limit Values Associated with Level 2 Environmental Characterization [63]
Ba	0.007	0.5
Cr	0	2
Mo	0	2.8
Ni	0.001	0.8
Cu	0.002	50
Zn	0.203	50
Cd	0	0.16
Hg	0.006	0.04
Pb	0	0.5
Sb	0	0.2
As	0	0.5
Se	0.007	0.4

**Table 5:** Mix design of the different mortars (dosages in kg/m^3^).

Constitutions	MCR-0%	MCR-10%	MCR-25%	MCR-50%	MCR-60%	MCR-75%	MCR-100%
Cement	400	400	400	400	400	400	400
Water	219	219	219	219	219	219	219
Superplasticizer	6	6	6	6	6	6	6
Limestone filler	300	300	300	300	300	300	300
Natural Sand (NS)	1328	1163	939	585	445	233	0
Crumb Rubber (CR)	0	47	117	234	281	351	427
Theoretical density	2253	2135	1980	1744	1651	1509	1352
W/C	0.55	0.55	0.55	0.55	0.55	0.55	0.55
W/B	0.31	0.31	0.31	0.31	0.31	0.31	0.31
r_v_ (%)	0	10	25	50	60	75	100
Density (kg/m^3^)	2241 ± 5	2121 ± 2	2030 ± 12	1815 ± 17	1763 ± 16	1692 ± 8	1447 ± 6

## Data Availability

The original contributions presented in the study are included in the article, further inquiries can be directed to the corresponding author.
